# The interplay of maternal and offspring obesogenic diets: the impact on offspring metabolism and muscle mitochondria in an outbred mouse model

**DOI:** 10.3389/fphys.2024.1354327

**Published:** 2024-03-22

**Authors:** Inne Xhonneux, Waleed F. A. Marei, Ben Meulders, Silke Andries, Jo L. M. R. Leroy

**Affiliations:** ^1^ Department of Veterinary Sciences, Laboratory of Veterinary Physiology and Biochemistry, Gamete Research Centre, University of Antwerp, Wilrijk, Belgium; ^2^ Department of Theriogenology, Faculty of Veterinary Medicine, Cairo University, Giza, Egypt

**Keywords:** DOHAD, intergenerational diseases, offspring health, obesogenic diet, maternal obesity, thrifty phenotype hypothesis

## Abstract

Consumption of obesogenic (OB) diets increases the prevalence of maternal obesity worldwide, causing major psychological and social burdens in women. Obesity not only impacts the mother’s health and fertility but also elevates the risk of obesity and metabolic disorders in the offspring. Family lifestyle is mostly persistent through generations, possibly contributing to the growing prevalence of obesity. We hypothesized that offspring metabolic health is dependent on both maternal and offspring diet and their interaction. We also hypothesized that the sensitivity of the offspring to the diet may be influenced by the match or mismatch between offspring and maternal diets. To test these hypotheses, outbred Swiss mice were fed a control (C, 10% fat, 7% sugar, and n = 14) or OB diet (60% fat, 20% sugar, and n = 15) for 7 weeks and then mated with the same control males. Mice were maintained on the same corresponding diet during pregnancy and lactation, and the offspring were kept with their mothers until weaning. The study focused only on female offspring, which were equally distributed at weaning and fed C or OB diets for 7 weeks, resulting in four treatment groups: C-born offspring fed C or OB diets (C » C and C » OB) and OB-born offspring fed C or OB diets (OB » C and OB » OB). Adult offspring’s systemic blood profile (lipid and glucose metabolism) and muscle mitochondrial features were assessed. We confirmed that the offspring’s OB diet majorly impacted the offspring’s health by impairing the offspring’s serum glucose and lipid profiles, which are associated with abnormal muscle mitochondrial ultrastructure. Contrarily, maternal OB diet was associated with increased expression of mitochondrial complex markers and mitochondrial morphology in offspring muscle, but no additive effects of (increased sensitivity to) an offspring OB diet were observed in pups born to obese mothers. In contrast, their metabolic profile appeared to be healthier compared to those born to lean mothers and fed an OB diet. These results are in line with the thrifty phenotype hypothesis, suggesting that OB-born offspring are better adapted to an environment with high energy availability later in life. Thus, using a murine outbred model, we could not confirm that maternal obesogenic diets contribute to female familial obesity in the following generations.

## Introduction

A sedentary lifestyle and consumption of Western-type diets [high-fat, high-sugar (HF/HS) diets] are associated with an increased worldwide prevalence of obesity, type II diabetes, and other metabolic disorders. According to Global Health Observatory (GHO) data, the prevalence of obesity in most countries is higher in women than men, with a poorer prognosis, imposing a major psychological and social burden on women (Bentley-Lewis et al., 2007; Westerman and Kuhnt, 2022). In addition, obesity in women is problematic as it not only impacts their own health and fertility but also increases the risk of metabolic disorders and obesity in the next generations, affecting future mothers and, eventually, the female germline (Eriksson et al., 2014; Contreras et al., 2016; Saben et al., 2016).

Currently, up to 40% of pregnancies in Western countries are to either overweight or obese mothers (Gaillard, 2015; Boutari and Mantzoros, 2022). Maternal BMI is positively associated with disease risk in adult offspring (Eriksson et al., 2014). This can be due to oocyte and embryo epigenetic alterations around conception and during pregnancy. However, whether such an increased risk is truly a direct consequence of maternal obesity is less clear because the family lifestyle is mostly persistent, and maternal dietary preferences and lifestyle are likely to influence children’s dietary choices (Gray et al., 2018; Pfledderer et al., 2021). Therefore, the increased risk of metabolic diseases in the offspring can be, at least in part, due to consuming an obesogenic (OB) diet in early life. Children who develop obesity at a young age are also more prone to the development of metabolic diseases later in life (Godfrey and Barker, 2000; Eriksson et al., 2014; Contreras et al., 2016; Stacy et al., 2019). In this context, the extent to which maternal obesity *per se* contributes to the continued propagation of the risk of obesity and metabolic disorders across generations and its interaction with the offspring’s response to a continued obesogenic diet are not clearly defined.

Multigenerational human studies are often confronted with difficulties linked to ethics, recruitment, randomization, equalization, drop-outs, and follow-up. Diet-induced obese mouse models circumvent some of these challenges and enable studying the underlying pathogenesis in a highly controlled setting (Brehm et al., 2010; Tuttle et al., 2018). The majority of studies in this field use inbred mice (most commonly the C57BL/6 strain). However, inbreeding is known to increase genetic drift, which may confound responses to environmental and nutritional stress (Nicholson et al., 2010). The C57BL/6 strain carries mutations in the nicotinamide nucleotide transhydrogenase (Nnt) gene, which may predispose them toward glucose intolerance (Freeman et al., 2006), and result in mitochondrial redox abnormalities (Ronchi et al., 2013). We tested and validated the use of the outbred Swiss mice to further increase the pathophysiological relevance to humans (Marei et al., 2020; Smits et al., 2021; Smits et al., 2022). In these studies, we showed that Swiss female mice fed a HF/HS diet exhibit obesity, hyperglycemia, hypercholesterolemia, and reduced insulin sensitivity. While maternal obesity has been shown to impact offspring health in murine inbred adult offspring (Samuelsson et al., 2008), this has not been tested in outbred mice.

In mice, HF diet-induced hypercholesterolemia and dyslipidemia are known to induce lipotoxicity in muscle tissue. Skeletal muscles account for 60%–70% of the insulin-stimulated glucose uptake and are described as a primary determinant of metabolic disorders (Turpin et al., 2009; Montgomery et al., 2017; Mikovic and Lamon, 2018). Lipotoxicity results in oxidative stress and mitochondrial dysfunction, with a reduction in oxidative phosphorylation capacity. This is associated with the impairment of insulin signal transduction pathways and reduced insulin sensitivity (Hauck and Bernlohr, 2016; Sergi et al., 2019).

While mitochondrial dysfunction is considered a key factor in the pathogenesis of metabolic diseases (Saris and Heymsfield, 2007; Sergi et al., 2019), mitochondria are also essential in intergenerational programming and the transmission of environmental effects to the next generations (Alhassen et al., 2021). Maternal obesity is directly linked with mitochondrial alterations, leading to placental dysfunction, modulating fetal growth and development (Sobrevia et al., 2020; Diniz et al., 2023), with gestational obesity directly affecting the offspring muscle metabolism (Walter and Klaus, 2014; Ampong et al., 2022). Previous research in inbred C57BL/6 mice suggested that ultrastructural aberrations in oocyte mitochondria induced by a maternal OB diet are transferred to the subsequent embryos. Mitochondria are exclusively maternally inherited. These aberrations were detected in oocytes and skeletal muscle tissues of offspring fed a standard chow diet but born to obese mothers (Saben et al., 2016). However, C57BL/6 mice have been found to already exhibit high rates of mitochondrial ultrastructural abnormalities in their oocytes even when fed a control diet when compared to outbred strains (Marei et al., 2020), which highlights the importance of the mouse strain used. Furthermore, the additive effect of offspring diet on these maternally induced effects on the offspring has never been investigated.

While most studies focus on the sole effect of maternal obesity on offspring health, Barker’s thrifty phenotype hypothesis states that fetuses are programmed to adapt to the environment they expect to be born in (Barker, 1997a; Godfrey and Barker, 2000). The interaction between maternal and offspring dietary effects is therefore critical, yet underexplored. Similar links between early-life adaptations and an individual’s phenotype development have been described (Saitou and Yamaji, 2012; Bateson et al., 2014). Hence, some of the reported effects of maternal obesity on offspring health might be due to the mismatch created by weaning offspring born to obese mothers on a control diet or *vice versa*. Understanding such interactions is important to develop tailored strategies to minimize the risks of metabolic diseases. This fundamental insight is crucial considering the current actual society needs (Liang et al., 2009; Sarker et al., 2019; Gawlinska et al., 2021).

Therefore, we hypothesized that 1) the offspring’s metabolic health is affected not only by direct exposure to an OB diet but also by the maternal dietary background. More importantly, we also hypothesized that 2) the offspring’s health is dependent on whether the maternal diet matches with the offspring’s diet. We aimed to test these hypotheses using an outbred Swiss mouse model in a 2 × 2 factorial design. Mothers were fed a control (C) or OB diet from 6 weeks before pregnancy until the end of lactation. After weaning, female offspring were fed either a C or OB diet. Effects on litter characteristics, offspring growth patterns, abdominal fat weight, blood profile, and mitochondrial features in oxidative skeletal muscle of female adult offspring were then evaluated and reported.

## Materials and methods

### Animal model and experimental design

**FIGURE 1:**
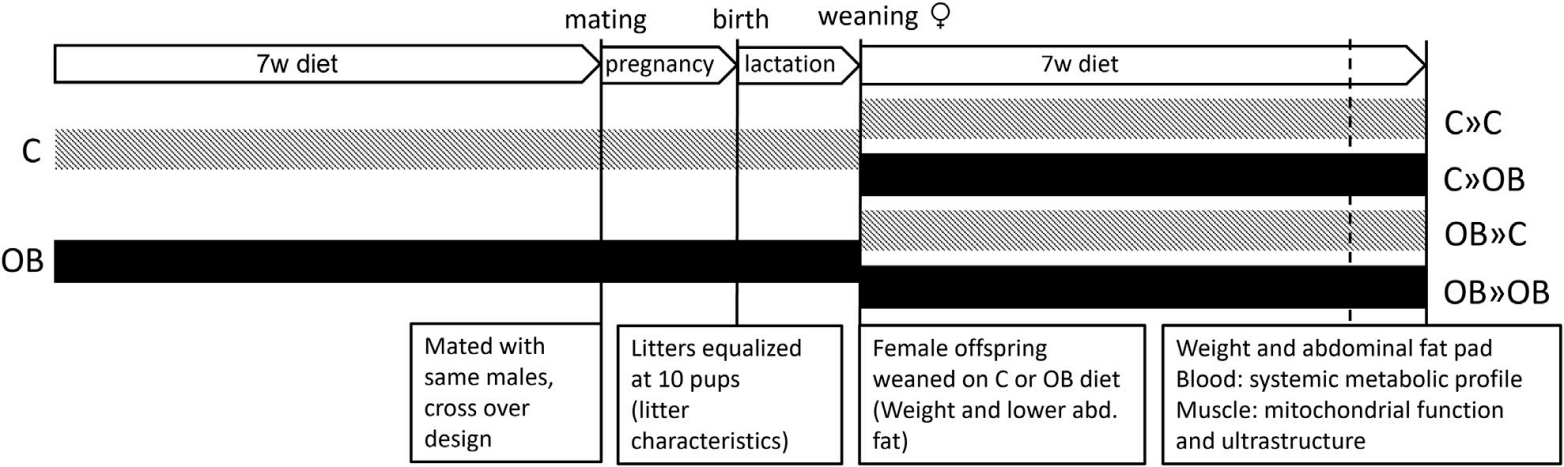
Schematic overview of the experimental design. Offspring were fed a control (C) or an obesogenic diet (OB) and were born to mothers that were either fed a C or OB diet in a 2 × 2 factorial design. The groups are named as MaternalDiet » OffspringDiet. This results in four experimental groups (C » C, C » OB, OB » C, and OB » OB).

**FIGURE 2:**
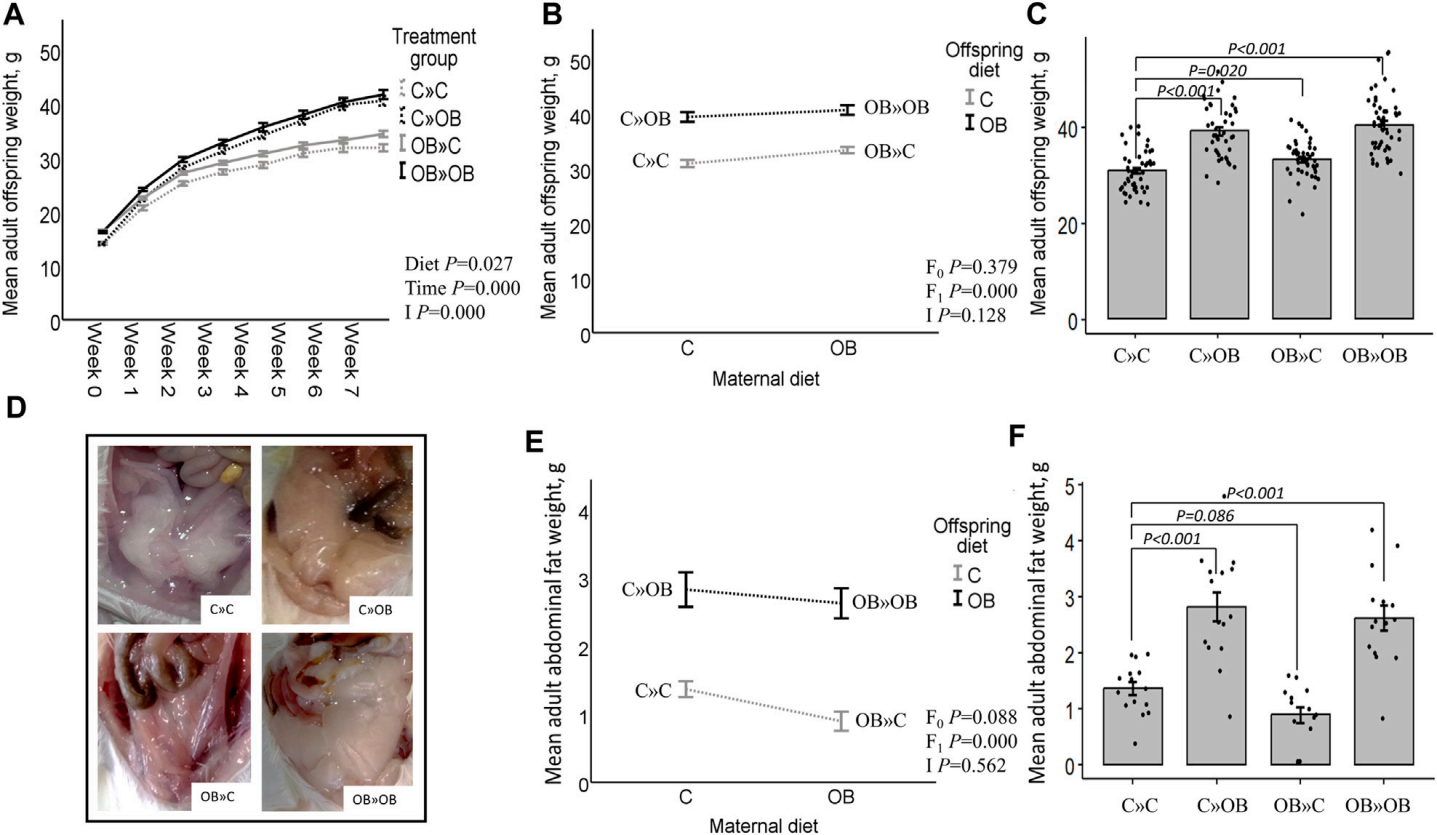
Maternal and offspring diet effects on adult offspring body weight and abdominal fat weight. Offspring growth curve **(A)**, interaction plot [two-way ANOVA, **(B)**] and bar chart with SE error bars and row data points [one-way ANOVA with C»C as reference group, **(C)**] of the mean abdominal fat weight (g) of offspring fed a C or an OB diet and born to mothers that were either fed a C or OB diet, in a 2 × 2 factorial design. Data are presented as mean±S.E.M and are derived from all offspring born to 6 C and 7 OB mothers. **(D)** Offspring abdominal fat. Interaction plot **(E)** and bar chart with SE error bars and row data points **(F)** of the mean adult abdominal fat weight (g) of all treatment groups. Data are presented as mean±S.E.M and are derived from all offspring born to 14 C and 15 OB mothers. *P*-values of the main effects are stated (F_0_ = maternal diet effect, F_1_ = offspring diet effect, I = interaction). *P*-values of significant differences and tendencies between treatment groups are displayed on the graphs.

This study was approved by the Ethical Committee for Animal Testing and performed accordingly (ECD 2018-05). In total, 14 female Swiss F_0_ mice were fed a control diet [C, 10% fat, 7% sugar, Sniff diets D12450J, containing 10 kJ% fat and 7% sucrose (E157453-04)], and 15 female Swiss F_0_ mice were fed an obesogenic diet [OB, E15741-34, 60% fat (beef Tallow), 20% sugar (fructose adjusted in the drinking water)] for 7 weeks. All females were mated (age 10 weeks) with the same Swiss males (n = 6) fed a standard chow diet in a cross-over design. Two days after birth, the litters were weighed, pups were counted, and their sex was recorded. Then, while there is conflicting evidence pro (Knight et al., 1986; Konig et al., 1988) and against (Enes-Marques and Giusti-Paiva, 2018; Briffa et al., 2019; Xavier et al., 2019) litter size normalization, we decided to equalize the litter size at 10 pups by only sacrificing male pups, allowing equal milk supply per pup and ruling out associated consequent effects on offspring health. As reported below, the litter characteristics were not affected by the diet. At 3 weeks, at least two female pups of six C and seven OB mothers were weighed and sacrificed to measure the weight of the lower abdominal fat (Tan et al., 2018). All other female offspring from each litter were weighed and equally weaned on a C or OB diet for 7 weeks, creating a 2 × 2 factorial study design (Figure 1) and resulting in four treatment groups named according to MaternalDiet » OffspringDiet: 1) C » C, pups born to C mothers (C-born) and fed a C diet (C-fed pups); 2) C » OB, C-born and OB-fed pups; 3) OB » C, OB-born and C-fed pups; and 4) OB » OB, OB-born and OB-fed pups. All pups were weighed weekly to record body weight trajectory (Figure 2). During the trial, several steps of stratification and randomization were applied to minimize the effects of potential confounders. As such, litters were randomly but equally weaned onto a C or an OB diet to have an equal number of sisters between different diet groups. In addition, pups of at least two mothers were grouped per cage, with an equal amount of total mice/cage, to avoid potential cage effects. Adult pups from six C and seven OB mothers were used to perform an insulin tolerance test (ITT) at 9 weeks of age after 6 h of fasting. After overnight fasting at 10 weeks of age, offspring from six C and seven OB mothers were sacrificed by decapitation, which was quickly performed by trained personnel using a sharp blade. Blood was collected for serum insulin, total cholesterol, triglyceride (TG), and alanine aminotransferase (ALT) analysis together with the abdominal fat, as described by Tan et al. (2018). Of these pups, one muscle (m. soleus) per pup per litter per group (nF_0_ = 6) was immediately snap-frozen in liquid nitrogen for later analysis of the expression of mitochondrial complex markers using Western blotting. The other m. soleus (nF_0_ = 5) was fixed in 2.5% glutaraldehyde solution for ultrastructural analysis using transmission electron microscopy (TEM). Offspring born to the other eight C and eight OB mothers were decapitated at 10 weeks of age, and blood was collected for non-esterified fatty acid (NEFA) analysis (Tor et al., 2021). The criteria to choose which animal was killed for each outcome parameter were random within nests [Excel, function = RAND ()] but practical between nests to allow blocking for the mother effect and father effect for all investigated outcome parameters.

### Assessment of the peripheral insulin response

All female offspring of six C and seven OB mothers were intraperitoneally injected with 0.75 IU insulin (Novo Nordisk, Denmark, Bagsværd, 0.15 IU/mL) per kg body weight after 6 h of fasting. Before the injection, basal glycemia was measured using a glucose meter and sticks from OneTouch Verio (LifeScan Belgium BV, Antwerp, Belgium) after a small tail cut. After injection, glycemia was measured after 15, 30, 60, 90, and 120 min. The area under the curve (AUC) and the elimination rate of glucose during the first 30 min after insulin injection (ER30) were calculated as described by Smits et al. (2021).

### Assessment of the systemic metabolic profile

The ultra-sensitive mouse insulin ELISA kit (Crystal Chem, Zaandam, the Netherlands) for low-range assays (0.1–6.4 ng/mL) was used on 15–16 offspring/group born to six C and seven OB mothers for the determination of insulin concentrations after overnight fasting. After equilibration, 95 µL of sample diluent was mixed with 5 µL of sample in antibody-coated microplate modules and incubated for 2 h at 4 °C. Samples were washed, and 100 µL of anti-insulin enzyme was added to stop the reaction; then, they were incubated for 30 min at room temperature (RT). Afterward, seven washing steps followed by a 40 min incubation of the enzyme-substrate solution (RT) were carried out, and the absorbance was measured with a TECAN Infinite M200pro microplate reader (TECAN Group Ltd., Männedorf, Switzerland). NEFAs (of eight offspring/group born to eight C and eight OB mothers) were colorimetric and enzymatic determined (Randox Laboratories, Crumlin, United Kingdom) in serum (A.M.L., Antwerp, Belgium) with a semi-automatic RX Monza analyzer for clinical chemistry assays (Randox Laboratories Ltd., Crumlin, United Kingdom; within CV 1%–3.1%, between CV 4.7%). ALT, TG, and cholesterol (of 10 offspring/group born to six C and seven OB mothers) were measured enzymatic (A.M.L., Antwerp, Belgium) on an Abbott Architect c16000 (Abbott, Illinois, United States; ALT within CV 2.34%, between CV 5.5%; triglycerides within CV 0.53%, between CV 2%; and cholesterol within CV 0.37%, between CV 2.5%) after 12 h overnight fasting. The analytical methodology of TG measurement is based on the reaction sequence described by Fossati and Prencipe (1982) and Mcgowan et al. (1983). The cholesterol detection is based on the formulation of Allain et al. (1974) and Roeschla et al. (1974). The catalyzing action of ALT transfers the amino group from L-alanine to 2-oxoglutarate in the presence of pyridoxal-5′-phosphate, forming pyruvate and L-glutamate. Pyruvate in the presence of NADH and lactate dehydrogenase is reduced to L-lactate, and in this reaction, NADH is oxidized to NAD. The rate of decrease in absorbance at 340 nm was monitored, as recommended by the International Federation of Clinical Chemistry (IFCC).

### Assessment of mitochondrial complex markers in muscle

Total OXPHOS Rodent WB Antibody Cocktail (Abcam, United Kingdom, Cambridge, ab110413) was used on the muscle tissue of six offspring/group born to six C and six OB mothers to analyze the expression of complex I, II, III, IV, and V markers in offspring oxidative muscle tissue. Housekeeping protein beta-actin antibody (ACTB, Cell Signaling Technology^®^, United States, Massachusetts, 4967S) was included to correct for differences in the sample load. The samples were lysed with RIPA buffer (300 µL/sample, Thermo Fisher Scientific™, Belgium, Dilbeek, 89900) combined with protease and phosphatase inhibitors (3 µL/sample, Thermo Fisher Scientific, 78425 and 1862495, respectively) followed by crushing (microtube homogenizer system, SP Bel-Art, Wayne, United States) and sonication (on ice, 6 × 10 s, and amplitude 50%) steps. The samples were centrifuged (5 min, 1,5000 rpm, and 4°C), and the supernatant was collected and heated (95°C) after adding Laemmli buffer (1/1) (Bio-Rad Laboratories, Belgium, Temse, 1610737) and Beta-mercaptoethanol (5%, Sigma-Aldrich^®^, United States, Missouri, M3148). SDS-page was performed on mini PROTEAN pre-cast gels (Bio-Rad, TGX Precast Protein Gels, 4561021) at 170 V and 0.5 A for 70 min. Afterward, proteins were transferred on PVDF membranes at 50 V and 0.1 A for 1 h 35 min and then blocked for 2 h in 0.1% skimmed milk (AppliChem GmbH, Germany, Darmstadt, A0830) and blotted overnight with an OXPHOS Rodent kit (dilution 1:500). Goat-anti-Mouse secondary antibody (dilution 1:2000, Agilent Dako Products, United States, California, P04448) HRP labeled and dissolved in 10% bovine serum albumin (Sigma-Aldrich^®^, S2002) solution containing 0.1% of NaN_3_ was used. In between different steps of blotting, the membrane was washed with TBST-Tween solution for 2 × 5 min, followed by 20 min of washing. To correct for differences in the protein load, ACTB was blotted on the same membrane after stripping in 20 mL 6.25 mM TRIZMA-base (pH 6.7, Sigma-Aldrich, United States, Missouri, T1503) containing 175 µL 2-mercaptoethanol (Sigma-Aldrich, M3148) on a rocking shaker for 30–45 min (RT). Protein bands were quantified with the Chemidoc XRS + System (Bio-Rad Laboratories).

### Assessment of mitochondrial ultrastructure in muscle

M. soleus of five offspring/group born to five C and five OB mothers was fixed upon collection in 0.1 M sodium cacodylate-buffered (pH 7.4) 2.5% glutaraldehyde solution, embedded in 2% agarose blocks, and washed in 0.1 M sodium cacodylate 7.5% saccharose solution (pH 7.4). Then, the blocks were incubated for 2 h in 1% OsO4 solution and dehydrated in an ethanol gradient. Ultrathin sections were cut using EM-bed812, stained with lead citrate, and examined with a transmission electron microscope, Tecnai G2 Spirit Bio TWIN microscope (Fei, Europe BV, Zaventem, Belgium), at 120 kV. Mitochondria were morphologically evaluated and classified by two researchers blind to the treatment group, according to the work of Hayden (2022). As morphological and functional differences occur between subsarcolemmal (SS) mitochondria and intermyofibrillar (IMF) mitochondria, these categories were investigated separately (Koves et al., 2005; Chomentowski et al., 2011). Mitochondria were classified as normal when they were homogenous without vacuoles, as described by Hayden (2022). Mitochondria with abnormal morphology contained large vacuoles (one large vacuole as described by Cook et al. (2014)), small dispersed vacuolization (characterized by enlarged intercristal spaces), as described by Hayden (2022), and other abnormalities such as the presence of mitochondrial-derived vesicle-like structures, as described by He et al. (2020), abnormal inner membrane formation, and mitochondria showing changed electron density.

### Statistical analysis

Statistical analysis was carried out using IBM SPSS Statistics 28 (for Windows, Chicago, IL, United States). Data were confirmed to be homogenous in variance (Levene’s test) and follow a normal distribution (residual QQ plots). Therefore, only parametric tests were used. Differences in maternal weight after 7 weeks of being fed their corresponding diet and maternal diet effects at birth and at weaning were analyzed using an independent-sample *t*-test. Correlations between maternal weight and litter characteristics were checked using Pearson’s correlations. Changes in maternal and offspring live body weight over time were compared using repeated measures ANOVA.

To test the effect of offspring diet, maternal diet, and their interaction at the age of 10 weeks (hypothesis 1), two-way ANOVA was used. In all the models, the maternal and the offspring diets were classified as the fixed factors. If two or more sisters were present in the same offspring treatment group, the mother ID was included as a random factor using linear mixed models. If the interaction was not significant, the interaction was omitted from the final model.

To estimate the effect of a match/mismatch between the maternal and the offspring diets (hypothesis 2), numerical data (e.g., abdominal fat and live body weight, blood analysis, and mitochondrial complex marker expression) were analyzed using one-way ANOVA (*post hoc* LSD correction) to compare the means and differences in the effect sizes of all treatment groups vs. C » C offspring as the healthy reference group.

Categorical data (e.g., proportions of abnormal mitochondrial ultrastructure in muscle tissue) were analyzed using binary logistic regression, testing both hypotheses in the same manner as the numerical data. Effect sizes were calculated using Cohen’s d coefficient and are shown separately in Figure 7. Subjective evaluations (e.g., mitochondrial ultrastructure) were independently generated by two experienced researchers blind to the corresponding treatment groups, and these data were merged after testing interrater reliability using the interclass correlation coefficient, as described by Koo and Li (2016).

Differences with *p*-values ≤ 0.05 are reported as statistically significant, and 0.05 < *p*-values ≤ 0.1 are reported as tendencies. All the main results are displayed in the manuscript and summarized in Supplementary Material S1. To account for the multiple comparisons performed in this study, we also included the corresponding q-values in Supplementary Material S1; Supplementary File S1.

## Results

### OB diet’s effects on maternal weight and litter characteristics at birth

Before looking at the offspring’s health parameters, we analyzed the effects of the OB diet on the mother’s weight and litter characteristics. Maternal OB diet increased the weight of the mothers at mating but had no effect on litter size, litter weight, or the sex ratio of the pups. The weight of the mother at the time of mating was also not correlated with litter size (r = 0.020). The original litter size was not correlated with adult offspring body weight (r = 0.974). Detailed information about the litter characteristics is included in Supplementary Material S2.

### Offspring live body weight and abdominal fat weight (weaning and post-weaning)

At weaning, maternal OB diet increased the offspring’s body weight and abdominal fat weight (detailed information is given in Supplementary Material S3).

After weaning, the offspring’s body weight trajectory was affected by time, the offspring’s diet, and their interaction. Maternal OB diet significantly increased offspring body weight at weaning and at adulthood. The maternal OB diet did not interact with the effect of the offspring’s diet. The offspring’s adult body weight was increased in all treatment groups compared to that of the reference group C » C.

Adult offspring’s abdominal fat weight was increased by the offspring’s OB diet and tended to be reduced by a maternal OB diet. The interaction of maternal and offspring diets was not significant. Adult offspring’s abdominal fat weight was increased in C » OB pups and OB » OB pups but tended to be reduced in OB » C pups compared to C » C.

Results of the offspring’s adult live body weight and abdominal fat weight are displayed in Figure 2.

### Offspring serum glucose and insulin

According to the two-way ANOVA analyses, the offspring’s OB diet significantly increased the AUC of the ITT and basal glucose and insulin levels, and it decreased ER30 rates, with no significant effect of or interaction with the maternal diet.

The effect of the offspring’s OB diet in increasing ITT (AUC) and basal glycemia and decreasing ER30 rate was evident in both C » OB pups compared to C » C. The effect on basal insulin was not significant in the one-way ANOVA comparison for this group. In the OB » OB group, basal glycemia was also significantly increased compared to C » C, with only a tendency of increased ITT (AUC), which could be due to the significantly higher basal insulin levels that were only detected in this group.

In contrast, while the maternal diet had no significant effect, the dietary mismatch in the OB » C was associated with a tendency to reduce the AUC of ITT and the basal glycemia compared to the controls.

**FIGURE 3:**
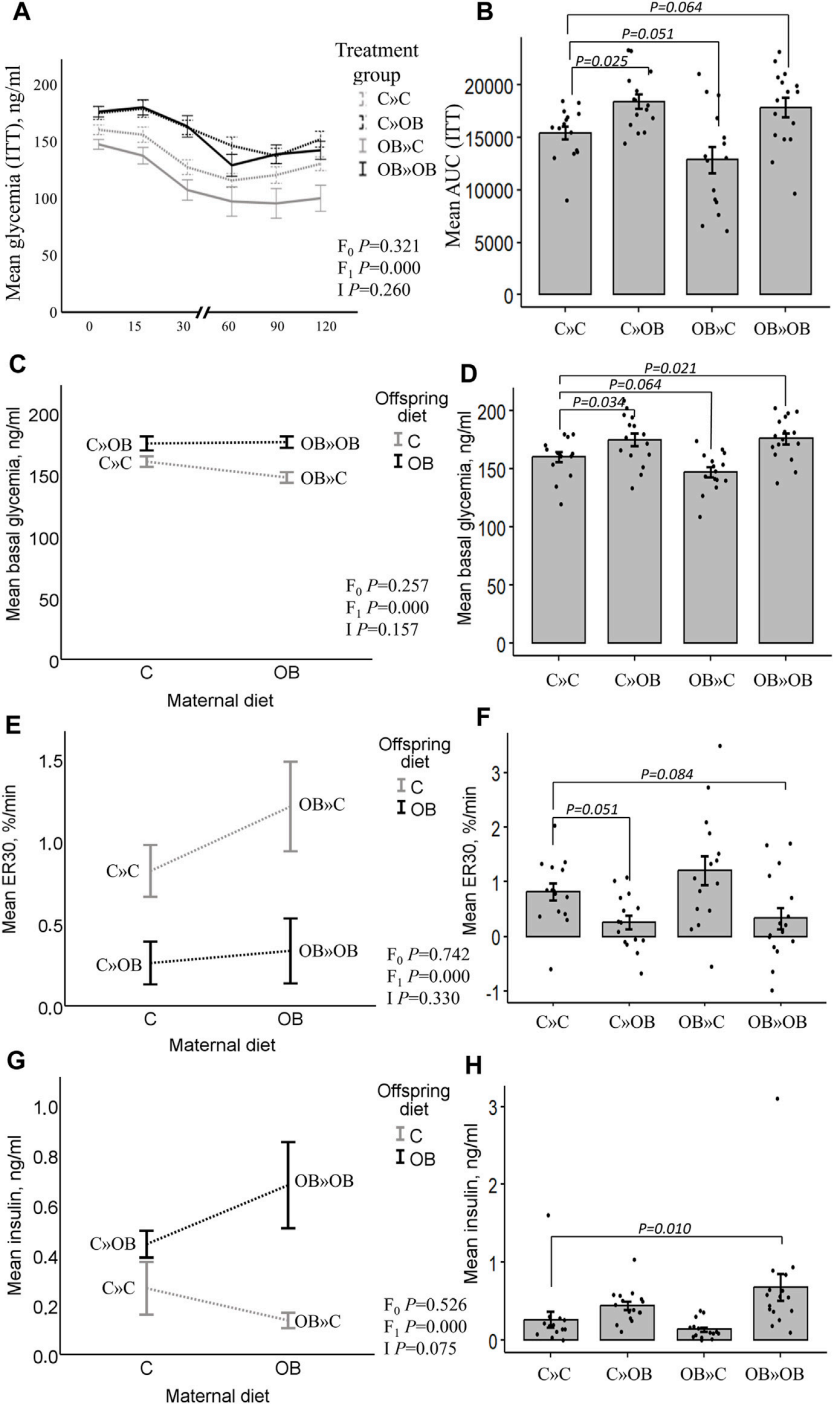
Maternal diet effects on offspring’s serum glucose profile and insulin concentrations. Graphs representing the mean glycemia [AUC (ITT)], **(A, B)**, the basal glycemia [**(C, D)**, ng/mL], the elimination rate during the first 30 min after insulin injection [ER30, **(E, F)**, %/min], and serum insulin [**(G,H)**, ng/mL] of offspring fed a C or an OB diet and born to mothers that were either fed a C or OB diet in a 2 × 2 factorial design. Data are presented as the mean ± S.E.M and are derived from 15–16 offspring/group born to six C and seven OB mothers. Interaction plots show two-way ANOVA analysis, and bar charts with SE error bars and row data points show one-way ANOVA comparisons with C » C as the reference group. *p*-values of the main effects are stated (F_0_ = maternal diet effect, F_1_ = offspring diet effect, and I = interaction). *p*-values of significant differences and tendencies between the treatment groups are displayed on the graphs.

Results of offspring serum glucose profile are displayed in Figure 3.

### Offspring serum lipid profile

Offspring serum cholesterol was significantly increased by the offspring’s OB diet, but it tended to be reduced by the maternal OB diet. The interaction was not significant. Serum cholesterol was significantly increased in C » OB offspring, but reduced in OB » C pups compared to C » C.

Offspring serum NEFA concentrations were only significantly increased by the offspring’s diet, with no effect or interaction of the maternal diet. This increase was only significant in the C » OB pups compared to C » C.

**FIGURE 4:**
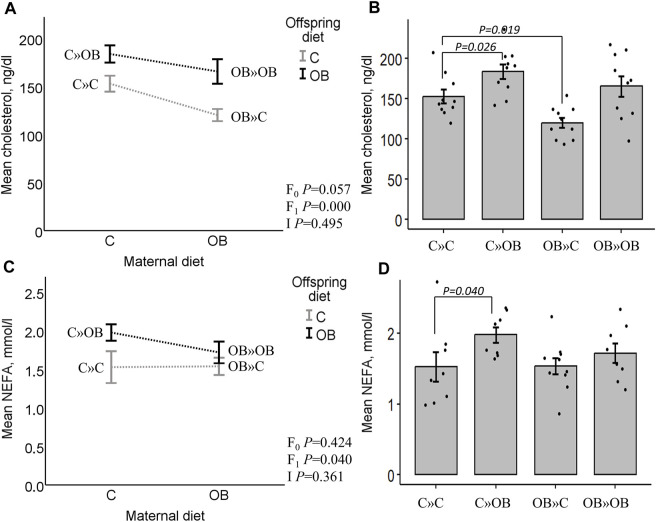
Maternal diet effects on offspring serum cholesterol and NEFA concentrations. Interaction plot **(A)** and bar chart **(B)** of mean serum cholesterol (ng/dL) from offspring fed a C or an OB diet and born to mothers that were either fed a C or OB diet in a 2 × 2 factorial design. Data are presented as the mean ± S.E.M and are derived from 10 offspring/group born to six C and seven OB mothers. Interaction plot **(C)** and bar chart **(D)** of the mean serum NEFA (mmol/L) of all treatment groups. Data are presented as the mean ± S.E.M and are derived from eight offspring/group born to eight C and eight OB mothers. Interaction plots show two-way ANOVA analysis, and bar charts with SE error bars and row data points show one-way ANOVA comparisons with C » C as the reference group. *p*-values of the main effects are stated (F_0_ = maternal diet effect, F_1_ = offspring diet effect, and I = interaction). *p*-values of significant differences and tendencies between treatment groups are displayed on the graphs.

Results of offspring serum cholesterol and NEFA levels are displayed in Figure 4.

Offspring serum ALT and TG were not affected by the offspring or maternal diet, or their interaction. No differences were detected between the groups (data are presented in Supplementary Material S4).

### Expression of mitochondrial complex markers in offspring m. soleus

In contrast with the systemic metabolic effects described above, significant maternal OB diet effects were detected at the muscle tissue level. Maternal diet increased the expression of mitochondrial complex markers III and V in m. soleus, with no effect of or interaction with the offspring’s diet. Interestingly, the pairwise comparison of the one-way ANOVA test showed a strong additive effect of the maternal and offspring diets on both complexes since OB » OB was the only significantly different group compared to C » C. Diet mismatch did not affect the complex marker expression. The expression of complex I, II, and IV markers was not affected by any of the examined factors.

**FIGURE 5:**
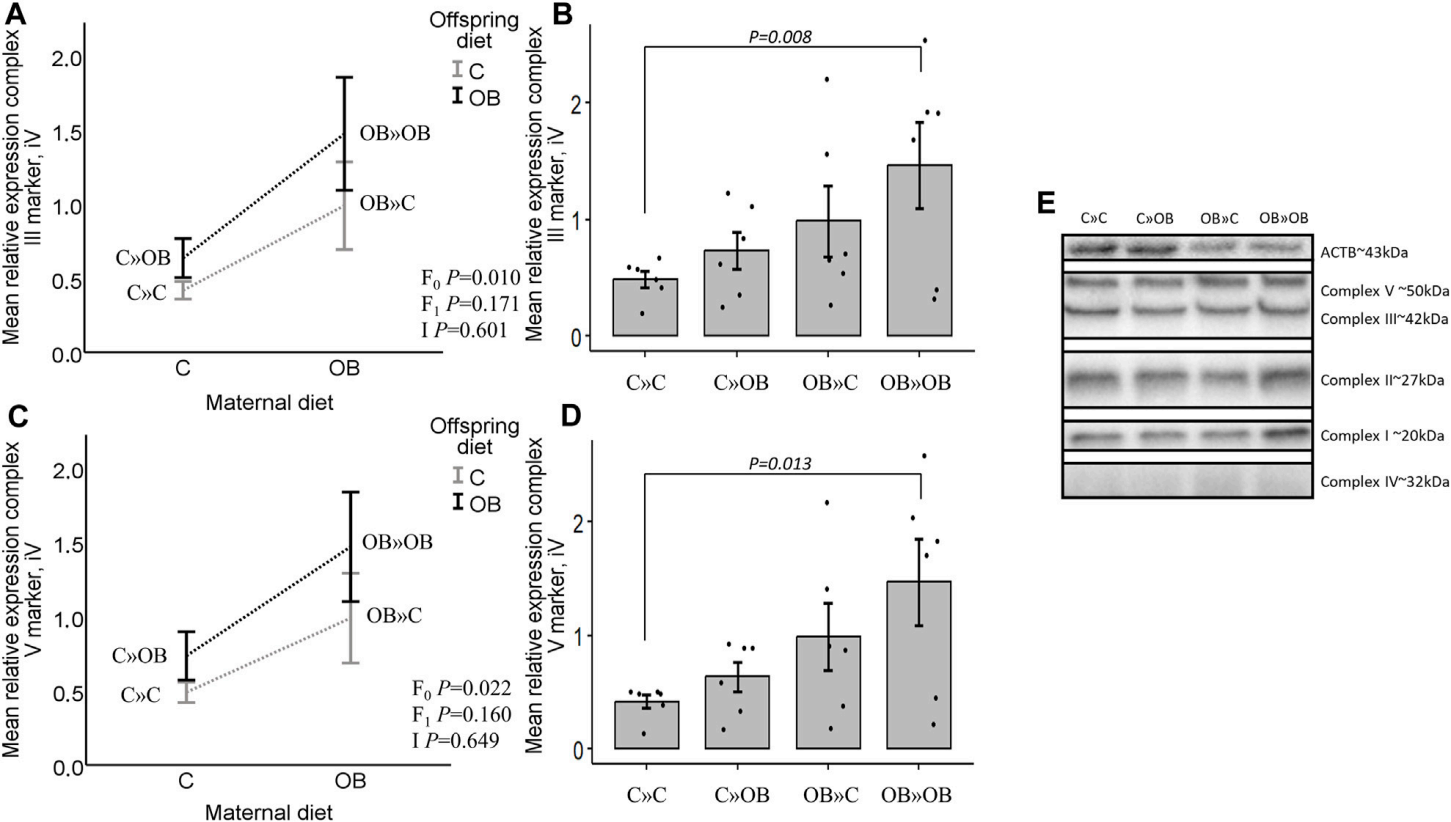
Maternal diet effects on offspring complex III and V marker expression. **(A)**. Graphs show the mean relative expression of complex III marker [**(A, B)**, iV] and complex V marker [**(C, D)**, iV] of offspring fed a C or an OB diet and born to mothers that were either fed a C or OB diet in a 2 × 2 factorial design. All data are presented as the mean ± S.E.M and are derived from six offspring/group born to six C and six OB mothers. Interaction plots show two-way ANOVA analysis, and bar charts with SE error bars and row data points show one-way ANOVA comparisons with C » C as the reference group. *p*-values of the main effects are stated (F_0_ = maternal diet effect, F_1_ = offspring diet effect, and I = interaction). *p*-values of significant differences and tendencies between the treatment groups are displayed on the graphs. **(E)**. PVDF membrane of one replicate, showing complex I, II, III, IV (increased exposure time), and V and housekeeping protein beta-actin (ACTB).

Results of mitochondrial complex markers III and V expression are shown in Figure 5. Results of mitochondrial complex markers I, II, and IV and PVDF membranes of all the samples are displayed in Supplementary Material S5.

### Offspring mitochondrial ultrastructure in m. soleus

The maternal diet also significantly reduced the proportion of morphologically normal mitochondria and increased the proportion of small and large vacuolization in both SS and IMF mitochondria in m. soleus. Interestingly, this maternal diet effect was highly dependent on the offspring’s diet (significant interaction). Offspring diet effects were mainly detected in IMF mitochondria but were less or not significant in SS mitochondria.

While the proportion of normal mitochondria was reduced in all the treatment groups compared to C » C, it is notable that the lowest values were observed in the offspring born to obese mothers but fed a mismatched control diet (OB » C). This group exhibited significantly higher rates of both small and large vacuolization in SS and IMF mitochondria. The dietary mismatch created in C » OB resulted in a different effect with only large but no small vacuolization. There was no additive effect of maternal and offspring diets noted (i.e., in OB » OB) since the rate of mitochondrial damage was comparable to that of other treatment groups.

**FIGURE 6:**
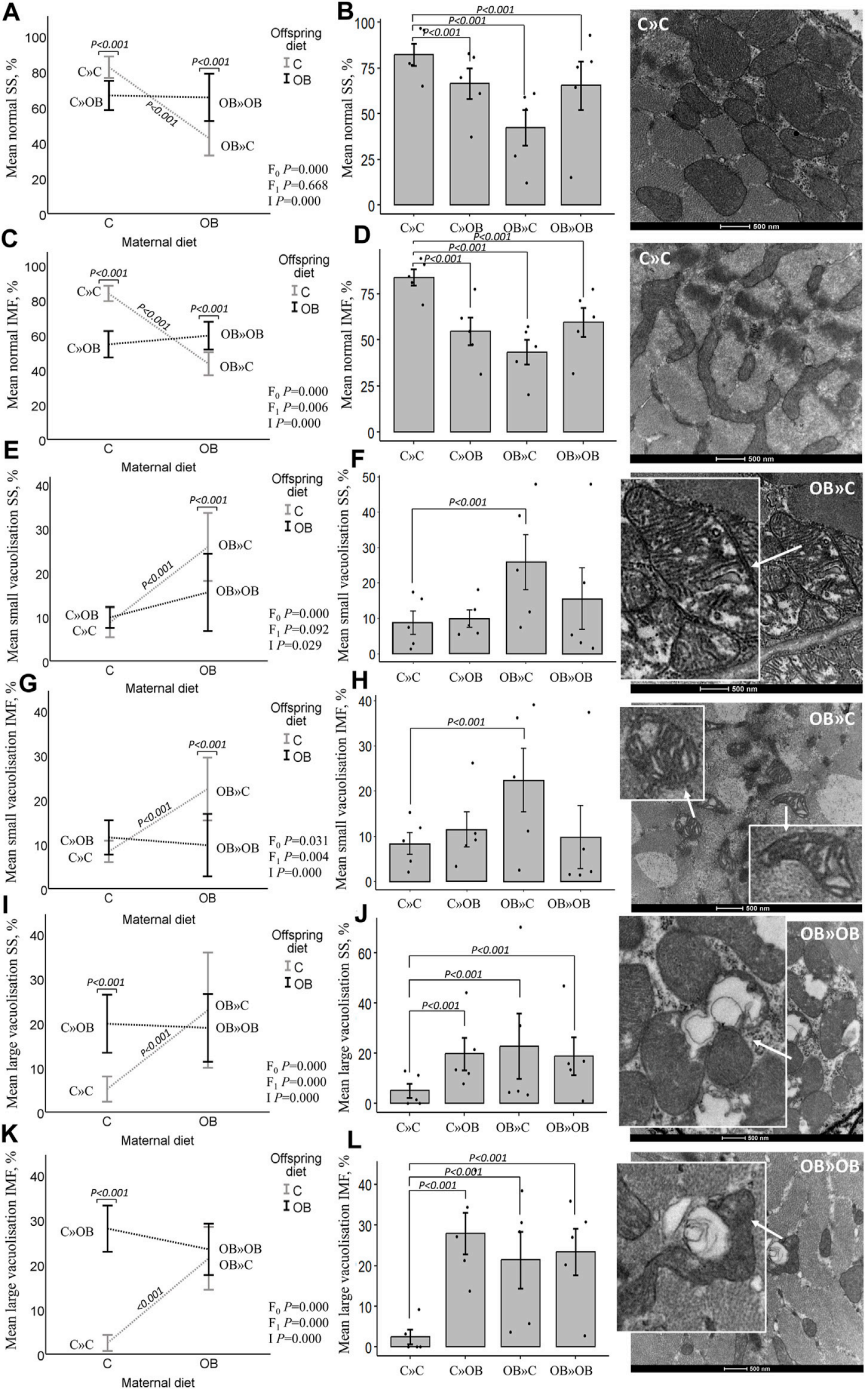
Maternal diet effects on offspring muscle SS and IMF mitochondrial ultrastructure. Mitochondrial ultrastructural morphology was classified and reported as homogenous SS **(A, B)** and IMF **(C, D)** mitochondria, small vacuolized SS **(E, F)** and IMF **(G, H)** and large vacuolized SS **(I , J)** and IMF **(K, L)** muscle mitochondria of offspring fed a C or an OB diet and born to mothers that were either fed a C or OB diet in a 2 × 2 factorial design. Data are presented using interactions plots and bar charts with SE error bars and row data points using binary logistic regression. Representative pictures for each mitochondrial phenotype are shown (right). All data are presented as the mean ± S.E.M and are derived from five offspring/group born to five C and five OB mothers. *p*-values of the main effects are stated (F_0_ = maternal diet effect, F_1_ = offspring diet effect, and I = interaction). *p*-values on the interaction plots represent pairwise comparison performed due to the significant interaction. *p*-values of significant differences between the treatment groups compared to C » C are displayed on the bar charts.

Results of offspring mitochondrial ultrastructure in m. soleus are displayed in Figure 6.

### Effect sizes

Finally, we provide an overview of the effect sizes of the different treatment groups compared to C » C in all the examined outcome measures. Notably, the effect of OB » C (denoted by orange triangles) is always distinct and sometimes opposite [in abdominal fat weight, ITT (AUC), basal glycemia, ER30, insulin, and cholesterol] from the other two groups (C » OB and OB » OB) in which the offspring diet effect is prominent. We also notice that the effect sizes of C » OB and OB » OB compared to C » C are mostly similar, showing no additive effects of maternal and offspring diets on the metabolic parameters or muscle mitochondrial features.

**FIGURE 7:**
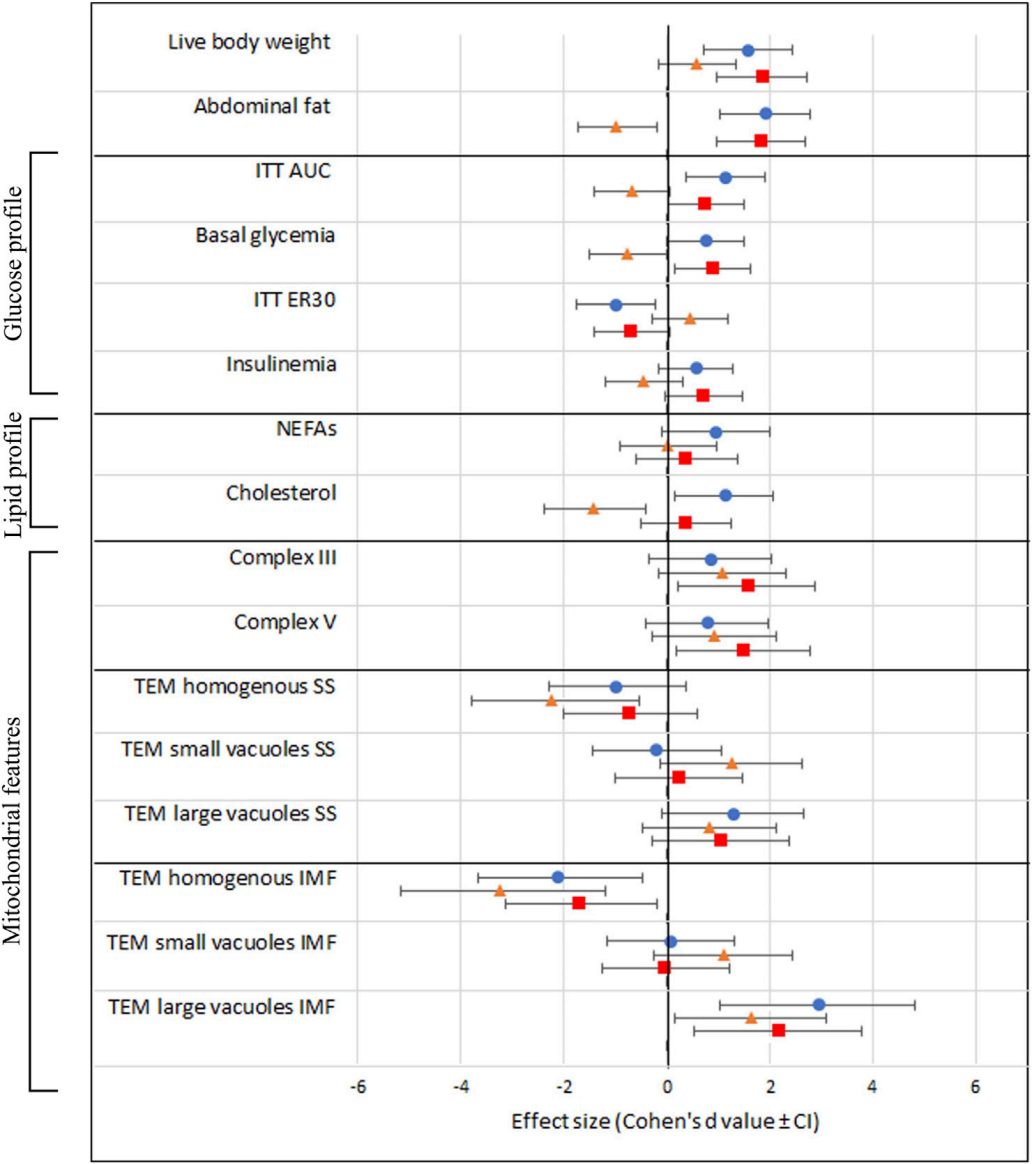
Schematic overview of all investigated outcome parameters with effect sizes estimated using Cohen’s d. The black vertical line represents the reference group C » C. Effect sizes are shown as Cohen’s d value ± CI and a blue dot (equals C » OB), orange triangle (equals OB » C), and red square (equals OB » OB).

The effect sizes are displayed in Figure 7.

## Discussion

We aimed to study the effect of maternal and offspring OB diets and their interaction on the offspring’s health and metabolism using an outbred mouse model. In addition, we aimed to examine if the dietary effects are influenced (attenuated or increased) by the match or mismatch between the maternal and offspring diets. This is the first study to perform such an intergenerational analysis in a 2 × 2 factorial design using an outbred mouse model, which increases the pathophysiological relevance in a setup that mimics the human situation of familial obesity. We chose to focus on female offspring health since the increased prevalence of obesity and metabolic diseases, particularly in women across generations, is alarming worldwide, with not only physical health effects but also significant psychological and social implications. In addition, while studying the same effects in male offspring in parallel can be of additive value, this would have doubled the data and significantly complicated the experimental design, data presentation, statistical comparisons, and discussion in this report. Therefore, this was practically not feasible.

The results we generated in C-born pups fed an OB diet (**C » OB *vs* C » C**) can be compared with the results of other studies investigating the direct effect of an OB diet in F_0_ female mice following standard breeding protocols. C » OB pups showed increased body weight and abdominal fat and significantly increased basal blood glucose levels. An increased AUC of the ITT and a slower clearance of glucose after insulin injection (ER30) indicate a reduced glucose tolerance due to an impaired sensitivity to insulin. Systemic glycemia reflects the balance of liver gluconeogenesis (starving or pre-prandial) and glucose uptake in the somatic tissue (Freeman et al., 2023). However, no liver functions were assessed in this study, and only general liver damage was estimated through ALT measurements. In addition, these pups also exhibited increased serum cholesterol and NEFA concentrations. Our data are in line with other studies reporting the direct response to OB diets in both inbred mice (male and female) (Wang and Liao, 2012; Vinué et al., 2018; Avtanski et al., 2019) and outbred mice (female) (Marei et al., 2020; Smits et al., 2021; Smits et al., 2022). This validates the model used in the present study. Fasting cholesterol levels were relatively high in the control mice but are in line with previous observations under the same experimental conditions (Marei et al., 2020).

Since mitochondrial dysfunction plays a key role in the development of reduced insulin sensitivity, our next step was to look at mitochondrial features in muscle tissue. Muscle tissue accounts for 60%–70% of the insulin-stimulated glucose uptake and is described as a primary determinant of metabolic disorders (Montgomery et al., 2017; Mikovic and Lamon, 2018; Sun et al., 2020; Smits et al., 2022). We noticed that the offspring OB diet-induced systemic alterations were associated with mitochondrial abnormalities in muscles. While the expression of the mitochondrial complex markers in the offspring’s muscle remained unaffected, the offspring’s OB diet clearly altered the offspring’s muscle mitochondrial morphology, with a significant increase in mitochondria with large vacuoles and a concomitant reduction in the percentage of normal mitochondria. These effects were different between the SS and IMF mitochondria since two-way ANOVA analysis showed a significant reduction of normal mitochondria only in IMF and not in SS mitochondria in response to the offspring’s diet. Koves et al. (2005) stated that SS and IMF mitochondria of the gastrocnemius muscle react differently to environmental changes due to increased uncoupling protein 3 content in SS mitochondria, a protein important in fatty acid metabolism, redox regulation, and ROS protection (Mailloux and Harper, 2011; Crescenzo et al., 2014). A human study performed by Chomentowski et al. (2011) showed that in m. vastus lateralis, only IMF, and not SS, mitochondrial volume density was correlated with BMI. In addition, IMF mitochondria were affected in patients with insulin resistance, whereas the SS mitochondria were not (Chomentowski et al., 2011). In our study, these differences were rather insubstantial.

Our main focus in this study was to investigate the maternal diet effects and whether the offspring diet’s effects were dependent on the maternal OB background. Maternal diet as a factor resulted in marked significant effects on muscle mitochondrial morphology and the expression of mitochondrial complex III and V markers. An interaction between the maternal and offspring diet effects was also significant (or tended to be significant) on basal insulin levels and muscle mitochondrial ultrastructural features. These results are in line with what has been previously described in studies using a similar experimental design (Saben et al., 2016; Dearden et al., 2020). However, in contrast, we were surprised that despite the major effects on muscle mitochondrial morphology, no maternal diet effects could be detected on adult offspring’s body weight, blood NEFA concentrations, and glucose homeostasis in response to insulin. We could even detect a tendency of reduced total blood cholesterol levels and abdominal fat in offspring born to OB mothers. This is all contradictory to what has been described in inbred mouse models (Samuelsson et al., 2008; Keleher et al., 2018). To further contemplate these results and to find out whether the sensitivity to the diet is influenced (attenuated or increased) by the match or mismatch between the maternal and offspring’s diet, it was necessary to study the differences and effect sizes in direct comparisons with the C » C mice as the most biologically healthy control group.

Focusing on the **OB » C** group, while these mice were slightly but significantly heavier than C » C, they had (or tended to have) a lower abdominal fat weight, lower serum cholesterol, reduced basal glycemia, and reduced AUC of glycemia during the ITT, with normal ER30 and mean insulin concentrations. So, an obesogenic maternal background only seems to result in a higher sensitivity to insulin and a better metabolic blood profile in the offspring fed a control diet after weaning. This is interesting since we expected the maternal OB diet to worsen the metabolic profile of the offspring (even when fed a C diet) instead of improving it, as described by others. Our results contradict the findings of Samuelsson et al. (2008), who associated a maternal OB diet with increased weight gain; increased risk of hyperglycemia, hypertension, and fatty liver disease; and reduced insulin sensitivity both in male and female pups. The latter study used inbred C57BL/6 mice, again highlighting the possible influence of the genetic background and inbreeding. It is also surprising that the normal (or slightly improved) metabolic profile in OB » C occurs while a significant reduction in the proportion of morphologically normal mitochondria was found in the m. soleus muscles. The mitochondria in the OB » C mice were specifically characterized by a significant increase in small vacuolization, with larger intercristal spaces. Increased intercristal space may be indicative of intra-organellar accumulation of fatty acids since Ho et al. (2002) and Schrauwen et al. (2010) stated that fatty acids can translocate into the mitochondrial matrix, bypassing acyl-coenzyme A synthase and carnitine palmitoyl acyl transferase I, resulting in the accumulation of fatty acids inside the mitochondria. This feature is observed without the occurrence of hyperlipidemia. Contrarily, the maternal OB diet tended to improve the offspring’s serum cholesterol in these mice. Such phenotype is similar to that described in the athlete paradox, where an increase in fat deposition in muscle tissue was an adaptive mechanism of muscles associated with a more efficient muscle energy production in highly trained athletes since these lipid droplets were used as a direct energy source and were not a consequence of hyperlipidemia (Goodpaster et al., 2001; Sergi et al., 2019). However, since muscle cellular respiratory functions, metabolic intermediates, lipid content, and lipid peroxidation levels were not analyzed in the present study, further investigations are required to test these notions at a functional level. In addition, since we show that increased mitochondrial ultrastructure abnormalities due to maternal diet effects are not detrimental to offspring health up to 10 weeks of age, studies using mitochondrial ultrastructural changes in muscle tissue as a functional indication or explanation of altered individual metabolic health should consider our results.

Last but not least, the metabolic profile and cellular characteristics of the **OB » OB** mice in the present model were also unexpected. Based on previous reports, we anticipated stronger synergetic effects due to the increased sensitivity to the OB diet in pups born to OB mothers. Keleher et al. (2018) showed that female offspring born to OB mothers (in inbred SM/J mice) and fed an OB diet had exacerbated obesity with increased abdominal fat weight compared to the offspring born to lean mothers and fed an OB diet. Interestingly, in the present study using outbred Swiss mice, the abdominal fat weight of OB » OB was similar to that of C » OB. The effect sizes induced by the OB » OB exposure on most of the investigated blood parameters were very similar, if not smaller, compared to the effects of C » OB compared with C » C (namely, on abdominal fat, ITT (AUC), basal glycemia, ER30, NEFAs, and cholesterol). In contrast with C » OB, the numerical increase in serum cholesterol and NEFA in OB » OB was not significant compared to C » C. Therefore, the matching OB » OB dietary exposure appears to result in a more favorable metabolic profile than the C » OB group. In addition to the strain differences, other factors may explain these contrasting results. For example, in the study of Keleher et al. (2018), pups were cross-fostered to lean mothers immediately after birth. In a study that cross-fostered C-born pups to obese mothers during lactation, Monks et al. (2018) suggested that nourishing from a mother consuming an OB diet may protect the offspring from the OB diet-induced effects later in life. Since we did not use cross-fostering here, trying to mimic the natural situation in humans in the best way possible, this may partially explain the differences.

Another interesting observation was that while there was a tendency of a maternal x offspring diet interaction (with *p* = 0.075) on blood insulin levels, elevated concentrations of insulin were only significant in the OB » OB mice compared to C » C. While this may indicate a more severe reduction in insulin sensitivity, effects on basal glycemia were similar to that in OB » C, and changes in glucose levels during the ITT (AUC) were even better (only a tendency compared to C » C). Thus, our results could not confirm a synergetic or aggravating effect of maternal and offspring OB diets on the offspring’s metabolic health parameters.

In addition, while the maternal diet had a significant effect on muscle mitochondrial complex III and V marker expression, this increase was again only significantly increased in the OB » OB group and not in the OB » C group compared to C » C. This is also opposite to what we expected because only a reduced expression of muscle mitochondrial complex I, II, IV, and V markers was associated with insulin resistance (Lee et al., 2021). Mitochondrial complexes are an assembly of multiple polypeptide chains arranged via functional intermediates and multiple chaperones (Vercellino and Sazanov, 2022). The electron transport system, including complex III, as well as complex V (ATP synthase) assembly and functions, are key elements in energy provision and cellular functions (Nakamoto et al., 2008; Nolfi-Donegan et al., 2020). The increased expression of complex III and V markers may be adaptive mechanisms to compensate for mitochondrial damage and improve mitochondrial energy production efficiency. We believe that these alterations at the cellular level are indicative of the adaptation of offspring towards an energy-rich environment. However, mitochondrial proteins can be regulated by post-translational modifications and super-complex assembly. Therefore, functional tests are still required to confirm these notions. Complex V assembly is also associated with changes in the mitochondrial ultrastructure (Jonckheere et al., 2012). Abnormal complex V dimerization (due to depleting specific subunits) may hamper normal cristae formation and result in onion-like structures (Paumard et al., 2002) similar to the features observed here in the mitochondria with large vacuoles, which were increased due to both the maternal and offspring diets. Mitochondria with large vacuoles containing loose inner membranes are also suggested by Cook et al. (2014) to be indicative of mitophagy, a temporary process by which damaged mitochondria are removed. Increased mitophagy is described as an adaptive response to improve insulin sensitivity (Ning et al., 2022). Future studies should include the outcome parameters focusing on mitophagy. While the effect on the proportions of these mitochondria was similar in OB » OB compared to C » OB vs. C » C, the magnitude (effect size) was smaller, and numerically more normal mitochondria were detected. In addition, the small vacuolization that was associated with the dietary mismatch in the OB » C group was not present in the OB » OB group. Mitochondrial shape, size, and cristae formation always adapt in response to changed energy requirements (Kondadi et al., 2019). Therefore, considering the metabolic profile associated with changes in mitochondrial morphology and complex III and V marker expression, OB » OB Swiss mice appear to perform better compared to C » OB.

The suggested changes in the OB » OB group may be adaptive and in line with the hypothesis of Holt (2023), stating that environmental developmental plasticity allows species to quickly adapt to changes over the following generations. Bateson et al. (2014) also indicated with the predictive adaptive response hypothesis that early life signals, predominantly stemming from the maternal environment, significantly influence an individual’s phenotype development, aiming at a better adaptation to similar environmental conditions later in life. The Dutch famine cohort study revealed that an increased prevalence of obesity was seen in offspring conceived during the Dutch famine of 1944–1945, showing that perturbations established during gestation may contribute to the development of offspring obesity in later life (Ravelli et al., 1999) and highlighting the importance of matching maternal and offspring diets. Vice versa, we believe that our data imply that the OB-born offspring are adapted to an energy-rich environment and, thus, ultimately support Barker’s theory of adaptational mechanisms toward changed nutrient availability and the thrifty phenotype hypothesis (Barker, 1997b).

We have previously shown that Swiss mice are responsive to the HF/HS diet, leading to an increase in weight gain, including hypercholesterolemia, and altered serum glucose (GTT) and insulin tolerance (ITT) after 7 weeks of feeding (Smits et al., 2021). In the present study, to avoid maternal stress before or during pregnancy that may interfere with the study results on the offspring’s metabolic profile (Enes-Marques and Giusti-Paiva, 2018; Sheng et al., 2023), we decided not to assess insulin sensitivity during gestation. Only maternal weight gain was recorded and shown to be significantly increased in OB mothers after 7 weeks compared to C-fed mothers. The offspring were sacrificed at 10 weeks of age, while often, older mice are used in studies focusing on insulin sensitivity and metabolic health (Fraulob et al., 2010; Ravichandran et al., 2019; Lee et al., 2021). Therefore, it is possible that offspring aging, coinciding with age-related mitochondrial dysfunction, may still affect or aggravate the mitochondrial features and the metabolic profile later in life. This may lead to more distinct differences between the treatment groups investigated in our study. In addition, in-depth intergenerational research may be needed to investigate the mitochondrial functions in muscle tissue of OB-born offspring to dissect the cause of the mitochondrial adaptation. Finally, since sex differences may occur in the development of metabolic disorders (Tramunt et al., 2020), a comparison between males and females can be of additive value in future intergenerational research to better understand the intergenerational impact on susceptibility.

In conclusion, this study is the first to investigate the effects of maternal and offspring OB diets and their interaction on the offspring’s metabolic health using an outbred Swiss mouse model. While the maternal diet was associated with abnormalities in muscle mitochondrial morphology, the effects of the maternal obesogenic background on the metabolic profile were very limited. On the contrary, offspring born to obese mothers and fed a normal diet seemed to have improved metabolic health during early adulthood. No additive effects (increased sensitivity) to an obese offspring diet were observed in pups born to obese mothers. In contrast, the metabolic profile appeared to be better than that of those born to lean mothers and fed an OB diet. These results are in line with the thrifty phenotype hypothesis, suggesting that OB-born offspring are better adapted to an environment with high energy availability later in life. These results differ from previous reports using inbred mice, highlighting the importance of the model when designing and interpreting intergenerational studies. Using a murine outbred model, we showed that maternal obesogenic diets as such do not embark upon female familial obesity in the following generations.

## Data Availability

The raw data supporting the conclusion of this article will be made available by the authors, without undue reservation.

## Abbreviations

HF/HS, high-fat, high-sugar; WHO, World Health Organization; OB, obesogenic; C, control; C » C, offspring born to a control mother and fed a control diet; C » OB, offspring born to a control mother and fed an obesogenic diet; OB » C, offspring born to an obesogenic mother and fed a control diet; OB » OB, offspring born to an obesogenic mother and fed an obesogenic diet; F_0_, maternal; F_1_, offspring; I, interaction; ITT, insulin tolerance test; AUC, area under the curve; ER30, elimination rate the first 30 min after insulin injection; NEFAs, non-esterified fatty acids; TG, triglyceride; ALT, alanine amino transferase; ROS, reactive oxygen species; SS, subsarcolemmal; IMF, intermyofibrillar; BMI, body mass index.
