# Supplementary material for: The interplay of maternal and offspring obesogenic diets: the impact on offspring metabolism and muscle mitochondria in an outbred mouse model
Source: Front Physiol. 2024 Mar 22;15:1354327. doi: 10.3389/fphys.2024.1354327 (PMC10995298; doi:10.3389/fphys.2024.1354327)
Supplement: Supplementary file 1 [file Table1.docx]

**The Interplay of Maternal and Offspring Obesogenic Diets:**

**Impact on Offspring Metabolism and Muscle Mitochondria in an Outbred Mouse Model.**

**Supplementary file 1. Main offspring postweaning results.**

Table S1.1. Post-weaning data of offspring fed a C or an OB diet and born to mothers that were either fed a C or OB diet, in a 2 x 2 factorial design. Data are shown as mean±S.E.M. Significant differences between treatment groups and C»C are shown by different letters (a, b). Tendencies are reported as dollar signs ($).

|  | C»C | C»OB | OB»C | OB»OB |
| --- | --- | --- | --- | --- |
| Body weight (g) | 24.38±1.03^a^ | 39.37±0.86^b^ | 33.48±0.56^b^ | 40.61±0.86^b^ |
| Abd. Fat weight (g) | 1.37±0.12^a^ | 2.83±0.25^b^ | 0.90±0.0.14^b$^ | 2.63±0.22^b^ |
| Cholesterol (ng/dl) | 152.9±8.3^a^ | 183.8±9.0^b^ | 120.2±6.3^b^ | 165.6±12.8^a^ |
| NEFA (mmol/l) | 1.53±0.21^a^ | 1.98±0.11^b^ | 1.54±0.11^a^ | 1.72±0.14^a^ |
| AUC (ITT) | 15451.5±644.0^a^ | 18435±702.3^b^ | 12870±1238.9^b$^ | 17861.3±919.7^b$^ |
| Basal glycemia (ng/ml) | 160.3±4.3^a^ | 175±5.6^b^ | 147.5±4.3^b$^ | 176.1±4.7^b^ |
| ER30 (%/min) | 0.8±0.2^a^ | 0.3±0.1^b^ | 1.2±0.3^a^ | 0.3±0.2^b$^ |
| Insulin (ng/ml) | 0.2±0.1^a^ | 0.5±0.1^a^ | 0.1±0.0^a^ | 0.7±0.2^b^ |
| C III (iV) | 0.42±0.06^a^ | 0.64±0.13^a^ | 0.99±0.29^a^ | 1.47±0.38^b^ |
| C V (iV) | 0.49±0.07^a^ | 0.74±0.16^a^ | 0.99±0.30^a^ | 1.47±0.37^b^ |
| Normal SS (%) | 83.32±6.07^a^ | 66.49±8.24^b^ | 42.35±9.70^b^ | 65.37±13.35^b^ |
| Normal IMF (%) | 83.80±4.37^a^ | 54.57±7.05^b^ | 44.45±6.61^b^ | 59.53±7.92^b^ |
| Small vac. SS (%) | 8.71±3.36^a^ | 9.85±2.39^a^ | 25.81±7.70^b^ | 15.53±8.75^b^ |
| Small vac. IMF (%) | 8.33±2.41^a^ | 11.50±3.86^a^ | 22.39±7.07^b^ | 9.74±7.02^a^ |
| Large vac. SS (%) | 5.15±2.85^a^ | 19.83±6.49^b^ | 22.90±12.97^b^ | 18.91±7.59^b^ |
| Large vac. IMF (%) | 2.47±1.79^a^ | 27.99±5.14^b^ | 21.69±7.02^b^ | 23.38±5.73^b^ |

Table S1.2. Corresponding q-value of post-weaning data of offspring fed a C or an OB diet and born to mothers that were either fed a C or OB diet, in a 2 x 2 factorial design. Data are shown as mean±S.E.M. Significant differences between treatment groups and C»C are shown by different letters (a, b). Tendencies are reported as dollar signs ($).

| Parameter | | Comparison | ***P*-value** | **q-value** | Parameter | Comparison | ***P*-value** | **q-value** |
| --- | --- | --- | --- | --- | --- | --- | --- | --- |
| Body weight | C»C - C»OB | | 0.001 | 0.048 | C III | C»C - C»OB | 0.549 | 0.561 |
| Body weight | C»C - OB»C | | 0.020 | 0.042 | C III | C»C - OB»C | 0.125 | 0.167 |
| Body weight | C»C - OB»OB | | 0.001 | 0.024 | C III | C»C - OB»OB | 0.008 | 0.020 |
| Abd. fat | C»C - C»OB | | 0.001 | 0.016 | C V | C»C - C»OB | 0.502 | 0.524 |
| Abd. fat | C»C - OB»C | | 0.086 | 0.118 | C V | C»C - OB»C | 0.180 | 0.216 |
| Abd. fat | C»C - OB»OB | | 0.001 | 0.012 | C V | C»C - OB»OB | 0.013 | 0.030 |
| AUC (ITT) | C»C- C»OB | | 0.025 | 0.046 | Normal SS | C»C - C»OB | 0.001 | 0.010 |
| AUC (ITT) | C»C - OB»C | | 0.074 | 0.108 | Normal SS | C»C - OB»C | 0.001 | 0.008 |
| AUC (ITT) | C»C - OB»OB | | 0.021 | 0.042 | Normal SS | C»C - OB»OB | 0.001 | 0.007 |
| B glycemia | C»C - C»OB | | 0.034 | 0.058 | Normal IMF | C»C - C»OB | 0.001 | 0.006 |
| B glycemia | C»C - OB»C | | 0.064 | 0.096 | Normal IMF | C»C - OB»C | 0.001 | 0.005 |
| B glycemia | C»C - OB»OB | | 0.021 | 0.040 | Normal IMF | C»C - OB»OB | 0.001 | 0.005 |
| ER30 | C»C - C»OB | | 0.051 | 0.079 | S vac SS | C»C - C»OB | 0.314 | 0.359 |
| ER30 | C»C - OB»C | | 0.168 | 0.218 | S vac SS | C»C - OB»C | 0.001 | 0.004 |
| ER30 | C»C - OB»OB | | 0.084 | 0.119 | S vac SS | C»C - OB»OB | 0.174 | 0.220 |
| Insulinemia | C»C - C»OB | | 0.256 | 0.300 | S vac IMF | C»C - C»OB | 0.038 | 0.063 |
| Insulinemia | C»C - OB»C | | 0.415 | 0.443 | S vac IMF | C»C - OB»C | 0.001 | 0.004 |
| Insulinemia | C»C - OB»OB | | 0.010 | 0.024 | S vac IMF | C»C - OB»OB | 0.178 | 0.219 |
| Cholesterol | C»C - C»OB | | 0.026 | 0.046 | L vac SS | C»C - C»OB | 0.001 | 0.004 |
| Cholesterol | C»C - OB»C | | 0.019 | 0.041 | L vac SS | C»C - OB»C | 0.001 | 0.003 |
| Cholesterol | C»C - OB»OB | | 0.345 | 0.385 | L vac SS | C»C - OB»OB | 0.001 | 0.003 |
| NEFA | C»C - C»OB | | 0.040 | 0.064 | L vac IMF | C»C - C»OB | 0.001 | 0.003 |
| NEFA | C»C - OB»C | | 0.958 | 0.958 | L vac IMF | C»C - OB»C | 0.001 | 0.003 |
| NEFA | C»C - OB»OB | | 0.368 | 0.401 | L vac IMF | C»C - OB»OB | 0.001 | 0.003 |

*B glycemia = basal glycemia, Abd. Fat = abdominal fat weight, S vac = small vacuolization, L vac = large vacuolization.*

| 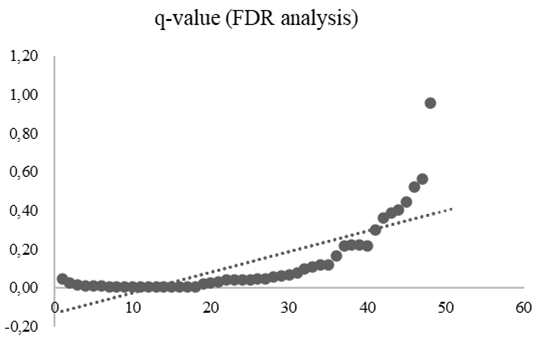 |
| --- |
| Figure S1.1 Q-values of post-weaning data of offspring fed a C or an OB diet and born to mothers that were either fed a C or OB diet, in a 2 x 2 factorial design. |
