# Supplementary figures and images for: The interplay of maternal and offspring obesogenic diets: the impact on offspring metabolism and muscle mitochondria in an outbred mouse model
Source: Front Physiol. 2024 Mar 22;15:1354327. doi: 10.3389/fphys.2024.1354327 (PMC10995298; doi:10.3389/fphys.2024.1354327)

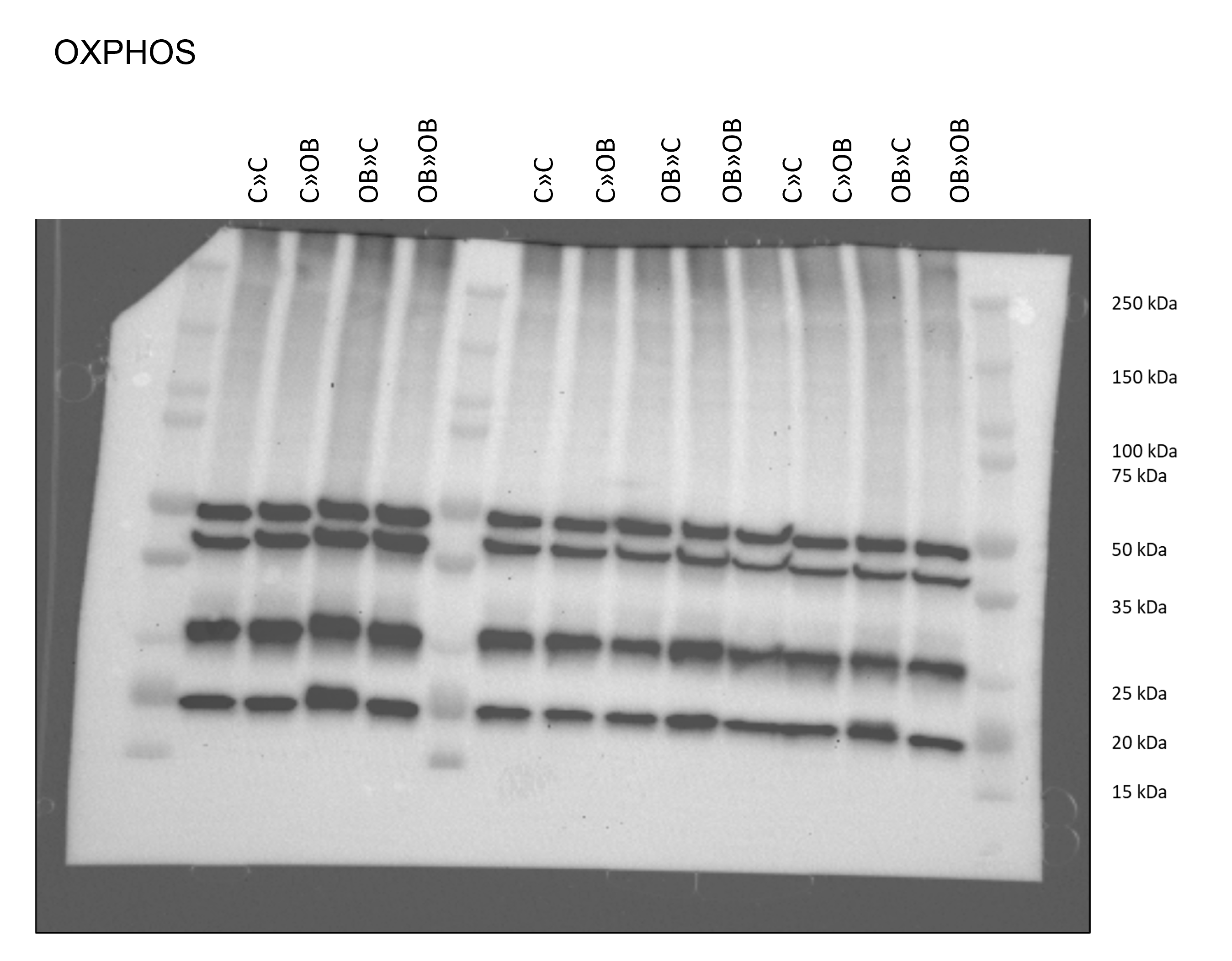

Supplement: Supplementary file 2 [file Image2.TIF]

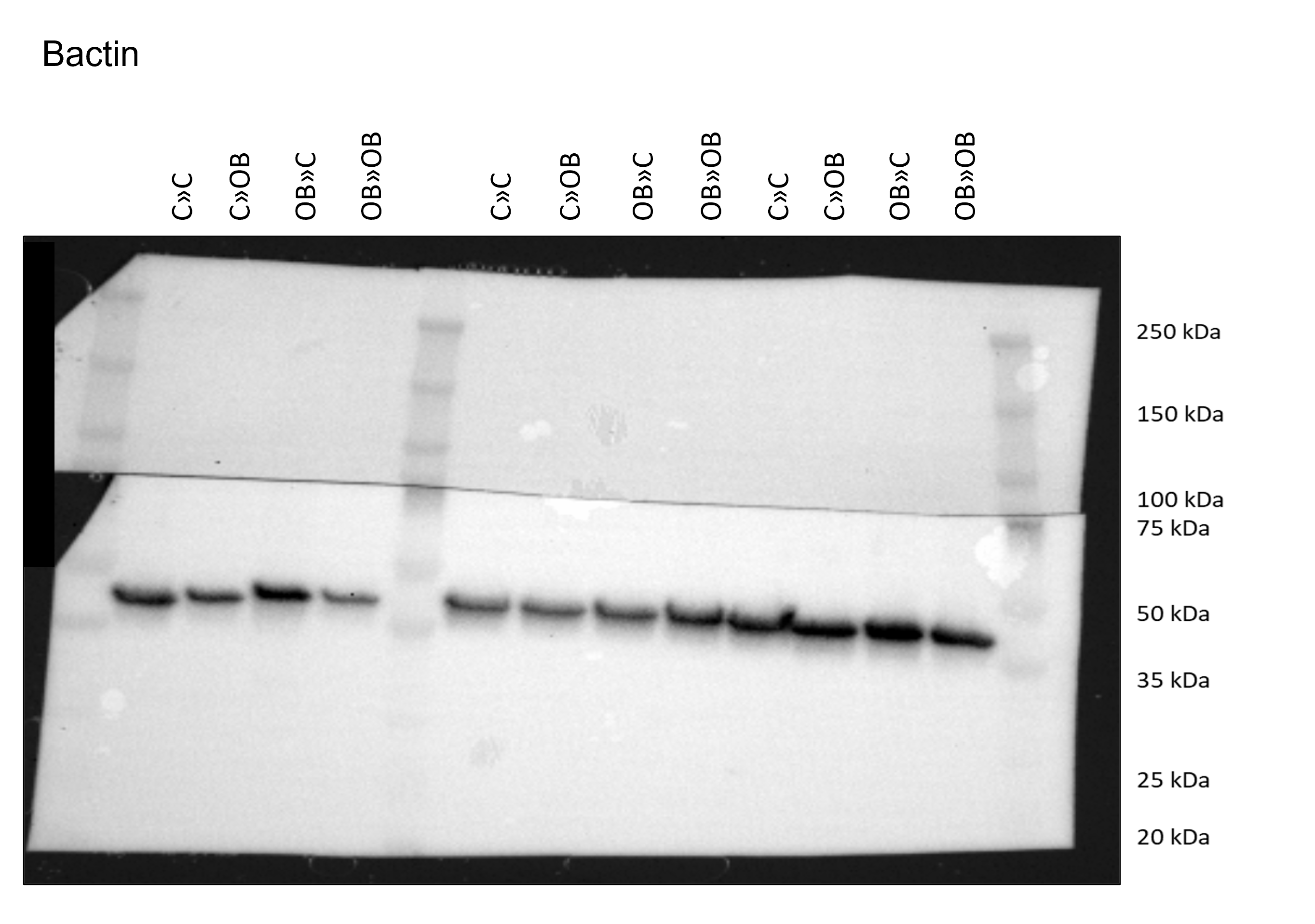

Supplement: Supplementary file 3 [file Image1.TIF]
