# Supplementary material for: The interplay of maternal and offspring obesogenic diets: the impact on offspring metabolism and muscle mitochondria in an outbred mouse model
Source: Front Physiol. 2024 Mar 22;15:1354327. doi: 10.3389/fphys.2024.1354327 (PMC10995298; doi:10.3389/fphys.2024.1354327)
Supplement: Supplementary file 4 [file Table2.docx]

**The Interplay of Maternal and Offspring Obesogenic Diets:**

**Impact on Offspring Metabolism and Muscle Mitochondria in an Outbred Mouse Model.**

**Supplementary file 2. Maternal live body weight and litter characteristics**

**Maternal live body weight**

During the trial, mothers were weighed weekly. Maternal live body weight significantly differed between C-fed mothers (32.97±1.24, g) and OB-fed mothers (41.15±1.69, g) after being fed their corresponding diet for 7 weeks (*P* < 0.001). The maternal growth curve is shown in figure S2.1, including the corresponding *P*-values for each timepoint.

| **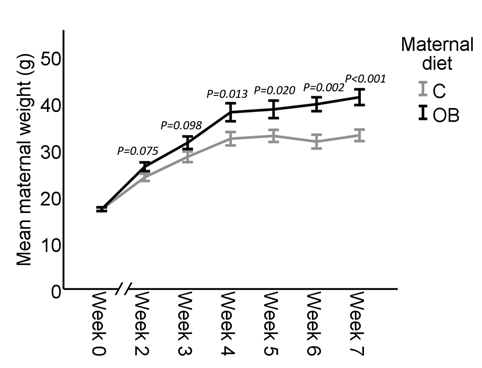** |
| --- |
| Figure S2.1. Maternal growth curve of mothers fed a C or an OB diet for 7 weeks. Data are shown as mean±S.E.M. and are derived from 14 C-fed and 15 OB-fed mothers. *P*-values of significant differences and tendencies are displayed on the graph. |

**Litter characteristics.**

Table S2.1. Litter characteristics of C- and OB- mothers, and their correlation with the maternal live body weight at mating. Data are shown as mean±S.E.M. The corresponding *P*- and r- values of the correlations are stated.

|  | Litter size | Litter weight (g) | Pup weight (g) | Female pups (%) | Male pups (%) |
| --- | --- | --- | --- | --- | --- |
| C mothers | 14.59±0.77 | 33.04±0.95 | 2.44±0.52 | 52.33±3.49 | 60.95±10.47 |
| OB mothers | 14.24±0.41 | 31.74±0.89 | 2.24±0.05 | 51.51±3.21 | 49.05± |
| *P*-value | 0.904 | 0.943 | 0.58 | 0.842 | 0.335 |
| r-value | 0.020 | 0.012 | -0.093 | 0.034 | -0.161 |

The original litter size was not correlated with adult offspring body weight (r = -0.109, *P* = 0.974).
