# Supplementary material for: The interplay of maternal and offspring obesogenic diets: the impact on offspring metabolism and muscle mitochondria in an outbred mouse model
Source: Front Physiol. 2024 Mar 22;15:1354327. doi: 10.3389/fphys.2024.1354327 (PMC10995298; doi:10.3389/fphys.2024.1354327)
Supplement: Supplementary file 5 [file Table5.docx]

**The Interplay of Maternal and Offspring Obesogenic Diets:**

**Impact on Offspring Metabolism and Muscle Mitochondria in an Outbred Mouse Model.**

**Supplementary file 5. Offspring muscle mitochondrial complex I, II and IV marker expression.**

| 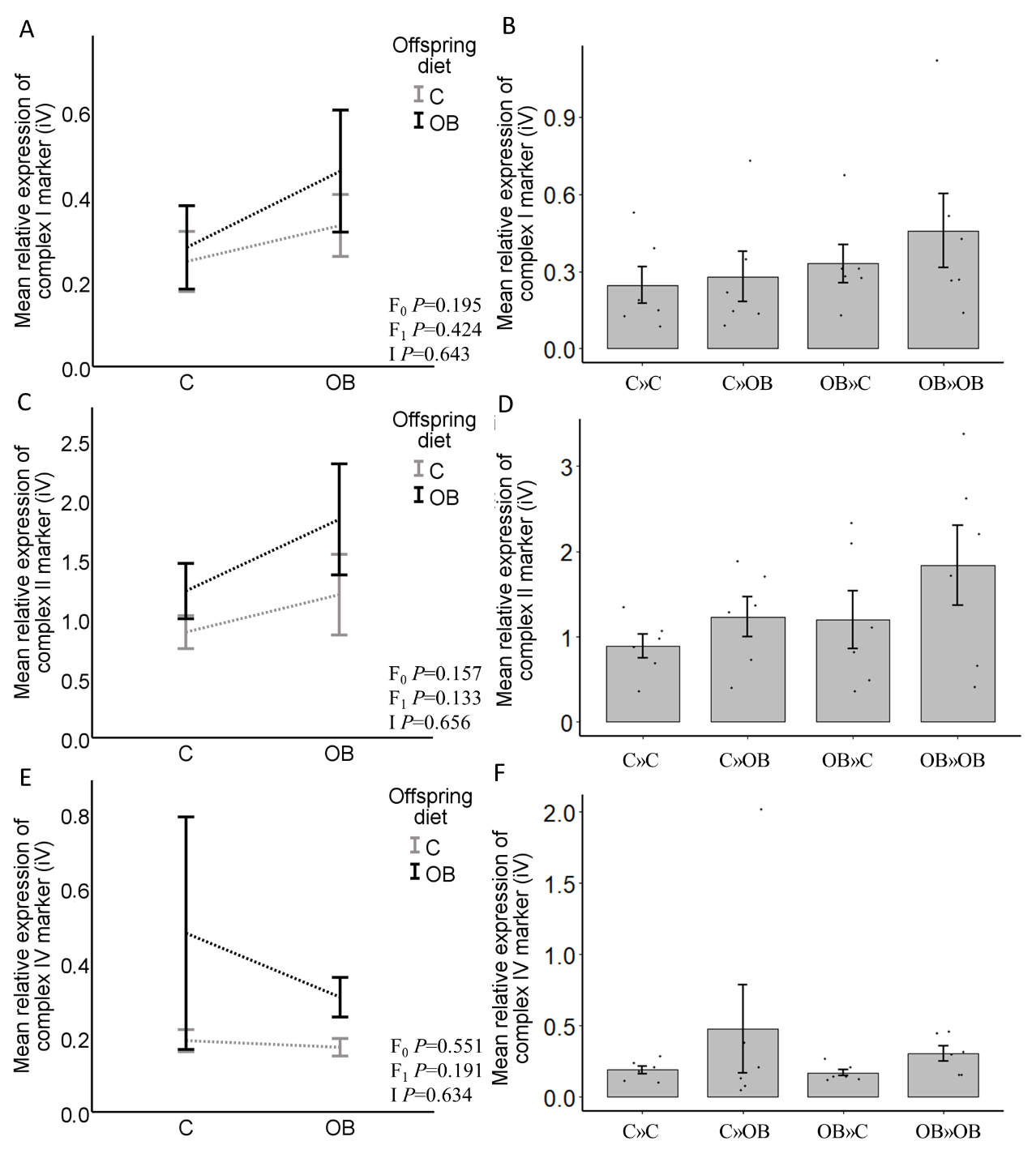 |
| --- |
| Figure S5.1. Maternal diet effects on offspring complex I, II and IV marker expression (muscle). A. Graphs show the mean relative expression of complex I marker (A, B, iV), complex II marker (C,D, iV) and complex IV marker (E,F) of offspring fed a C or an OB diet and born to mothers that were either fed a C or OB diet, in a 2 x 2 factorial design. All data are presented as mean±S.E.M and are derived from 6 offspring/group born to 6 C and 6 OB mothers. Interaction plots show Two-way ANOVA analysis, bar charts with SE error bars and row data points show One-way ANOVA comparisons with C»C as reference group. *P*-values of the main effects are stated (F_0_ = maternal diet effect, F_1_ = offspring diet effect, I= interaction). |

| 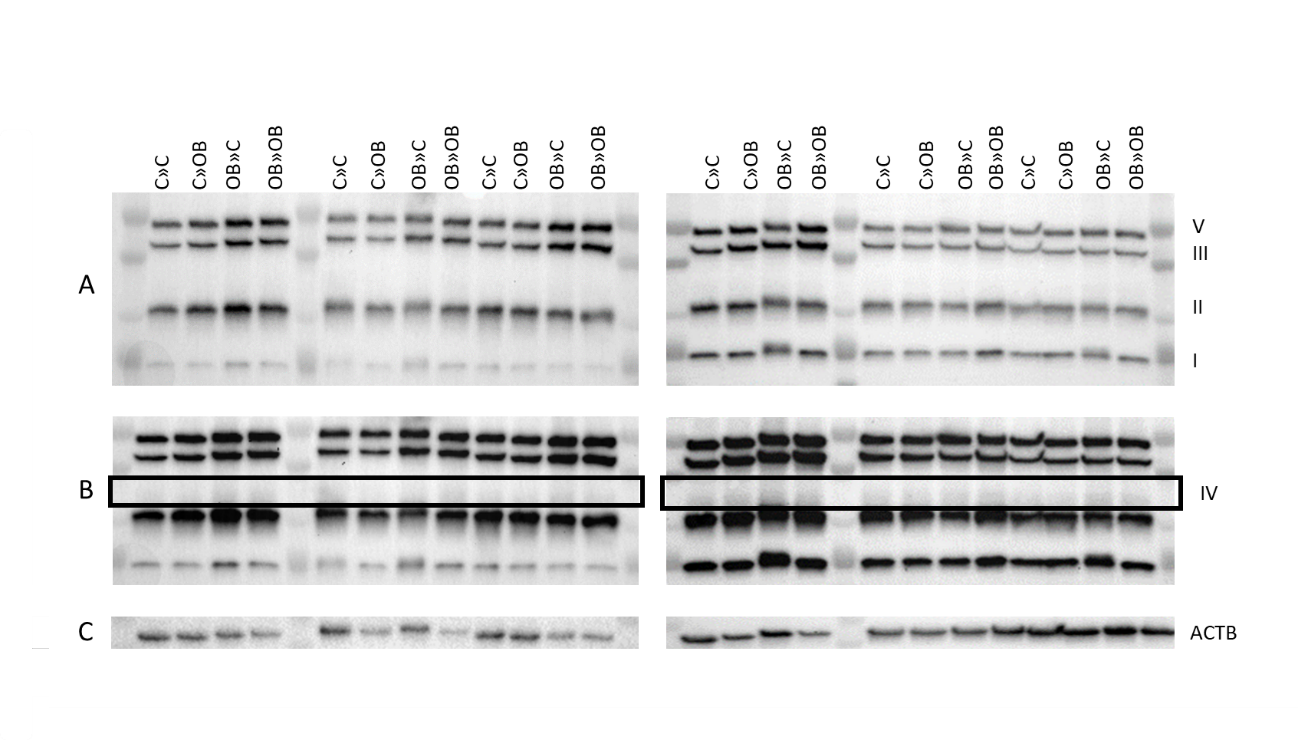 |
| --- |
| Figure S5.2. PVDF-Membrane of all replicates, showing complex I, II, III, IV (increased exposure time) and V, and housekeeping protein Beta-actin (ACTB). A. PVDF-membrane for measuring mitochondrial complex I, II, III and V expression. B. PVDF-membrane prolonged exposure (290s) for measuring complex IV expression. C. PVDF-membrane for measuring Beta-actin (ACTB) as housekeeping protein after stripping, to correct for differences in sample load. |
