# Supplementary material for: The interplay of maternal and offspring obesogenic diets: the impact on offspring metabolism and muscle mitochondria in an outbred mouse model
Source: Front Physiol. 2024 Mar 22;15:1354327. doi: 10.3389/fphys.2024.1354327 (PMC10995298; doi:10.3389/fphys.2024.1354327)
Supplement: Supplementary file 6 [file Table3.docx]

**The Interplay of Maternal and Offspring Obesogenic Diets:**

**Impact on Offspring Metabolism and Muscle Mitochondria in an Outbred Mouse Model.**

**Supplementary file 3. Offspring body weight and abdominal fat weight at weaning, and offspring body weight trajectory after weaning.**

**Offspring body weight and abdominal fat weight at weaning**

Table S3.1. Pre-weaning data of offspring body weight and abdominal fat weight of offspring born to C- and OB- mothers. Data are shown as mean±S.E.M. Significant differences are shown by different letters (a, b). Tendencies are reported as dollar signs ($).

|  | C-born pups | OB-born pups |
| --- | --- | --- |
| Body weight (g) | 14.02±0.67^a^ | 16.14±0.15^b^ |
| Weight abdominal fat (g) | 0.06±0.01^a^ | 0.12±0.01^b^ |

| 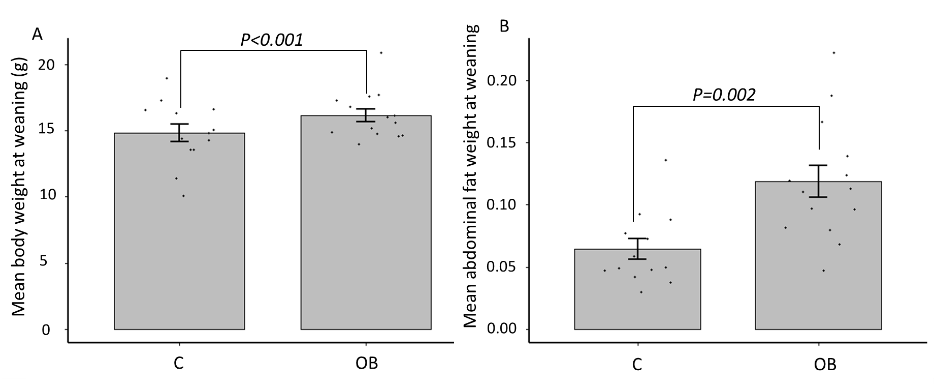 |
| --- |
| Figure S3.1. Maternal diet effect on offspring live body weight and abdominal fat weight at weaning. Bar chart with SE and row data points of mean offspring live body weight (A, g) and offspring abdominal fat weight (B, g) at weaning. Data are presented as mean±S.E.M and are derived from at least 2 offspring born to 6 C and 7 OB mothers. Corresponding *P*-values are displayed on the graph. |

**Offspring body weight trajectory after weaning**

After weaning, offspring body weight trajectory was affected by time (*P* = <0.001), offspring diet (*P* = 0.027), and their interaction (*P* < 0.001). Maternal OB diet significantly increased offspring body weight at week 1 (*P* < 0.001), wk2 (*P* = 0.001) , wk3 (*P* = 0.006) and wk4 (*P* = 0.083) post-weaning, but this effect disappeared from 5 wk onwards. The effect of the offspring OB diet was already significant at wk1 post-weaning and was further significant until sample collection at wk7 (10 weeks of age). During this growth trajectory, the maternal OB diet never interacted with the effect of the offspring diet.
