# Supplementary material for: The interplay of maternal and offspring obesogenic diets: the impact on offspring metabolism and muscle mitochondria in an outbred mouse model
Source: Front Physiol. 2024 Mar 22;15:1354327. doi: 10.3389/fphys.2024.1354327 (PMC10995298; doi:10.3389/fphys.2024.1354327)
Supplement: Supplementary file 7 [file Table4.docx]

**The Interplay of Maternal and Offspring Obesogenic Diets:**

**Impact on Offspring Metabolism and Muscle Mitochondria in an Outbred Mouse Model.**

**Supplementary file 4. Offspring serum alanine aminotransferase (ALT) and triglycerides (TG).**

| 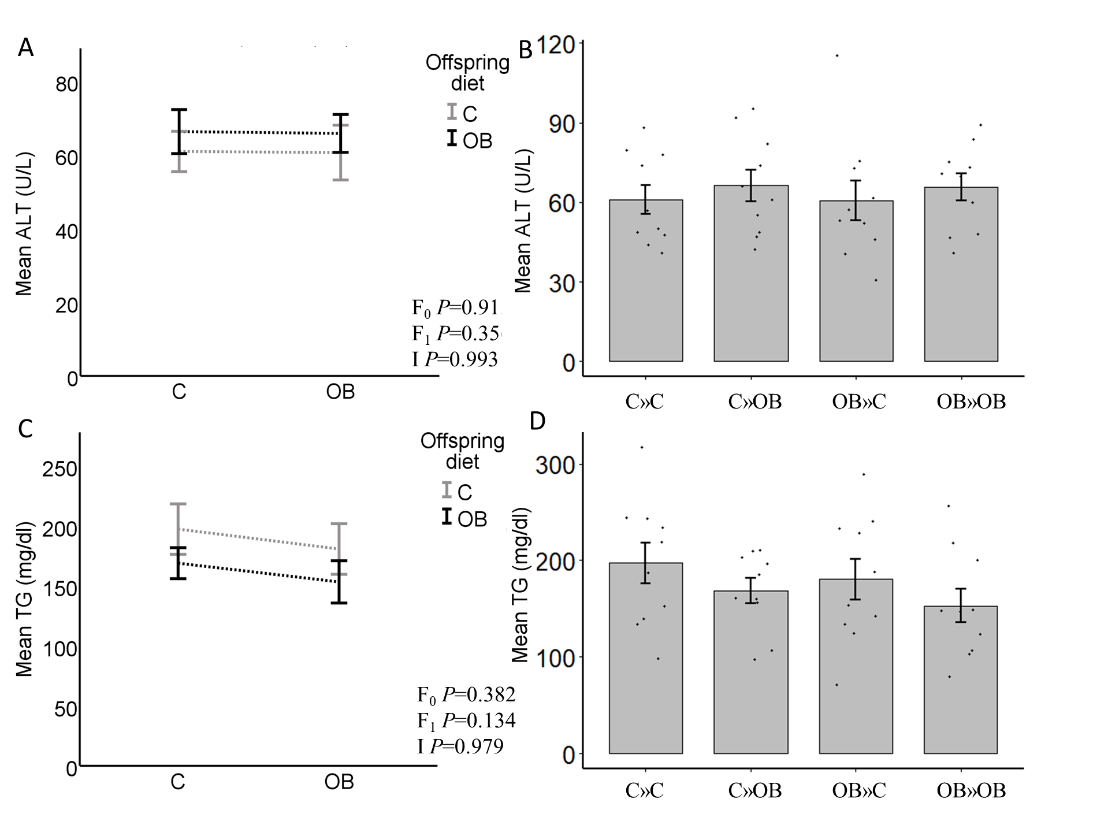 |
| --- |
| **Figure S4.1**. Maternal diet effects on offspring serum ALT and TG concentrations. Interaction plots (A,C) and bar chart (B, D) of mean serum ALT (U/L) and TG (mg/dl) from offspring fed a C or an OB diet and born to mothers that were either fed a C or OB diet, in a 2 x 2 factorial design. Data are presented as mean±S.E.M and are derived from 10 offspring/group, born to 6 C and 7 OB mothers. Interaction plots show Two-way ANOVA analysis, bar charts with SE error bars and row data points show One-way ANOVA comparisons with C»C as reference group. *P*-values of the main effects are stated (F_0_ = maternal diet effect, F_1_ = offspring diet effect, I= interaction). |
